# Carrion crows (*Corvus corone*) of southwest Germany: important hosts for haemosporidian parasites

**DOI:** 10.1186/s12936-017-2023-5

**Published:** 2017-09-12

**Authors:** Sandrine Schmid, Katrin Fachet, Anke Dinkel, Ute Mackenstedt, Friederike Woog

**Affiliations:** 1 University of Hohenheim, Emil-Wolff-Straße 34, 70599 Stuttgart, Germany; 2 State Museum of Natural History Stuttgart, Rosenstein 1, 70191 Stuttgart, Germany

**Keywords:** Avian malaria, Multiple infections, *Pica pica*, *Plasmodium*, *Haemoproteus*, *Leucocytozoon*, Corvidae

## Abstract

**Background:**

Avian malaria parasites (*Plasmodium* spp.) and other Haemosporida (*Haemoproteus* and *Leucocytozoon* spp.) form a diverse group of vector-transmitted blood parasites that are abundant in many bird families. Recent studies have suggested that corvids may be an important host for *Plasmodium* spp. and *Leucocytozoon* spp.

**Methods:**

To investigate the diversity of Haemosporida of resident carrion crows (*Corvus corone*) and Eurasian Magpies (*Pica pica*) in southwest Germany, 100 liver samples of corvids were examined using a nested PCR method to amplify a 1063 bp fragment of the haemosporidian mitochondrial cytochrome *b* gene. The phylogenetic relationship of parasite lineages obtained from these birds was inferred.

**Results:**

Haemosporidian DNA was detected in 85 carrion crows (89.5%) and in all five Eurasian Magpies. The most abundant parasite genus was *Leucocytozoon* with a prevalence of 85.3% (n = 95). 65.3% of the samples (n = 62) contained multiple infections. Thirteen haemosporidian lineages were isolated from the corvid samples. Female carrion crows were more likely infected with haemosporidian parasites than males.

**Discussion:**

This study provides the first insight into the diversity of haemosporidian parasites of corvids in Germany. Very high prevalences were found and based on the applied diagnostic method also a high amount of multiple infections could be detected. Due to the high diversity of haemosporidian parasites found in corvids, they seem to be excellent model organisms to test species deliminations in haemosporidian parasites.

## Background

Avian malaria parasites (*Plasmodium* spp.) and other Haemosporida (*Haemoproteus* and *Leucocytozoon* spp.) form a diverse group of vector-transmitted blood parasites that are abundant in many avian families [1]. All haemosporidian parasites share similar but complex life cycles. They use blood-feeding dipterans as vectors. *Plasmodium* species are known to be transmitted by several species of mosquitoes (Culicidae) from different genera, *Haemoproteus* species by several species of hippoboscid and ceratopogonid flies and *Leucocytozoon* species are known to be transmitted by simuliid and ceratopogonid flies [2]. The sexual reproduction occurs in the gut of the vector and the infectious sporozoites develop in their salivary glands. When the vector feeds on a vertebrate host, the sporozoites enter the blood stream and invade hepatocytes or endothelial cells [3] where the parasites then undergo the first cycle of asexual schizogony. Once released, the merozoites infect new cells of various tissues where they undergo another cycle of asexual schizogony. In contrast to most other avian haemosporidians, *Plasmodium* parasites also undergo schizogony in erythrocytes. During an acute infection, the merozoites within erythrocytes or leucocytes develop into gametocytes, which can then infect a new vector. Avian haemosporidian parasites can also develop dormant stages which remain inactive in the tissues. After a latent stage of infection, a period of chronic parasitaemia, a secondary increase of parasitaemia can occur due to an reactivation of exoerythrocytic merogony [1, 4].

Avian haemosporidian parasites have been detected worldwide except Antarctica (e.g. [1, 5, 6]). It has been suggested that the species diversity of Haemosporida may be at the same level of avian species diversity [7] or even higher.

The bird family Corvidae consists of more than 100 species of crows (*Corvus* spp.), jays (e.g. *Aphelocoma* spp., *Calocitta* spp., *Cyanocitta* spp.), magpies (*Pica* spp.) and allies, has an almost worldwide distribution and is present in almost all habitats. Past studies of haemosporidian parasites in Corvidae relied mostly on morphological examination of blood smears (e.g. [8–10]). According to Valkiunas [1], two *Haemoproteus* species (*Haemoproteus danilewskii* and *Haemoproteus picae*) and two *Leucocytozoon* species (*Leucocytozoon berestneffi* and *Leucocytozoon sakharoffi*) are the most important Haemosporida species for corvids. There seems to be no *Plasmodium* species that exclusively infects corvids. The MalAvi database [11] relies on published sequences of haemosporidian parasites from different bird hosts. According to this database, 98 different haemosporidian lineages have been isolated from different corvid species (as of May 2017). For carrion crows (*Corvus corone*) 14 haemosporidian lineages have been found [12–15], whereas for Eurasian Magpies (*Pica pica*) only two *Haemoproteus* sequences are published [16].

Recent studies have suggested that corvids may be an important host for *Plasmodium* spp. [17, 18] and *Leucocytozoon* spp. [19, 20]. However, these studies are based on investigations of only a few individuals. Clearly, more data are needed.

Carrion crows and Eurasian Magpies are widespread breeding birds in central Europe and ringing recoveries show a high natal and breeding site fidelity [21]. Except for very few young individuals that disperse, most birds are resident and do not show migratory behaviour. Especially in urban areas their number has increased and they have become synanthropic [19].

The purpose of our study was to assess the presence and to investigate the diversity of haemosporidian parasites of carrion crows (*Corvus corone*) and Eurasian Magpies (*Pica pica*) in southwest Germany. For parasite detection, a highly sensitive nested PCR was used to amplify a fragment of 1063 bp of the parasite mitochondrial cytochrome *b* gene. Phylogenetic analyses and the construction of a haplotype network allowed untangling the relationship of parasite lineages obtained in this study. Furthermore, it was tested, whether the age or sex of a bird has an effect on the prevalence of haemosporidian parasites. It was predicted that (1) older birds have a higher prevalence of blood parasites due to higher exposure time and (2) females have a higher prevalence than males due to reduced locomotion during breeding and the presence of a brood patch.

## Methods

### Origin and preparation of samples

In accordance with the German legislation 100 corvids (95 carrion crows (*Corvus corone*) and five Eurasian Magpies (*Pica pica)*) were shot in August and September 2016 in course of a predator management programme in southwest Germany. Sample sites were located in a 25 km radius around Stuttgart (48°47′N; 9°11′O). The birds were frozen at −20 °C until further examination.

Plumage and bill colour were used for aging carrion crows into juvenile (<2 years; n = 31), second calendar-year (n = 33) or adult (>2 years; n = 31) [22] and sex was determined by identifying the reproductive organs (42 males, 49 females). Due to severe damages after shooting, the age and sex of the remaining birds could not be identified.

Tissue samples were taken from the heart and liver and transferred into 1.5 µl Eppendorf tubes containing 200 µl of DPBS (Dulbecco’s Phosphate Buffered Saline; Sigma-Aldrich Chemie GmbH, Munich, Germany). Tissue samples are deposited at the collection of the State Museum of Natural History (Stuttgart).

Morphological examination of blood using blood smears was not possible as birds were shot and frozen directly. During freezing and subsequent thawing the blood cells rupture, thus the identification of intracellular parasites is not possible anymore.

### Parasite screening using PCR

DNA was extracted using the Zymo Research extraction kit (Quick-gDNA™ MiniPrep; Zymo Research Europe GmbH, Freiburg, Germany) according to the manufacturer’s instructions but with the following modifications: the lysis of tissue was increased by adding 20 µl of Proteinase K in the first step and the samples were then incubated at 60 °C for 10 min. After extraction DNA was stored at −20 °C until further use. Initial molecular screening of the heart and liver samples gave identical results. Therefore, only liver tissue was used in successive tests except in rare cases of unclear results or failed tests whereby analysis was rerun using heart tissue.

Target sequence for amplification was a 1063 bp fragment of the haemosporidian mitochondrial cytochrome *b* gene. For amplifying such a long fragment, a nested PCR was developed using the primers listed in Table 1. To control possible contamination with target DNA, a negative control was included in each test run as well as a positive control to ensure PCR was working properly.

**Table 1:** Primers used for amplification of haemosporidian cytochrome *b* gene

PCR (5′–3′)	nested PCR (5′–3′)
CytF1: GCCTAGACGTATTCCTGATTATCC	CytFN: GCTTTAAATGGTTGGAATATG
CytR1: CATAATTATAACCTTACGGTCTG	CytRN: GTTTGCTTGGGAGCTGTAATC
HaemNF: CATATATTAAGAGAATTATGGAG^a^	HaemF: AATGGTGCTTTCGATATATGCATG^b^
HaemNR2: AGAGGTGTAGCATATCTATCTAC^a^	HaemR2: GCATTATCTGGATGTGATAATGGT^b^
	LeucoF: CCCATTCGTAGCATTAGC

^a^Previously designed by [23]

^b^Previously designed by [24]

The PCR amplifying the full fragment was conducted in two steps. The primer pair CytF1 and CytR1 was used to amplify a 1332 bp fragment in the first PCR and the internal primer pair CytFN and CytRN was used to amplify a 1224 bp fragment in the second PCR.

The reaction mixture consisted of 10 mM Tris–HCl, 50 mM KCl, 2 mM MgCl_2_, 20 pmol of each primer, 200 μM of each dNTP, 1.25 units Ampli-Taq (Applied Biosystems, Carlsbad, USA) and for the first PCR approximately 10–100 ng DNA in a total volume of 50 μl. For the second PCR 2 μl of the amplification product was used as template. Both PCRs were performed for 40 cycles with each cycle consisting of denaturation at 94 °C for 30 s, annealing at 52 °C for 30 s and elongation at 72 °C for 90 s.

**Fig. 1:**
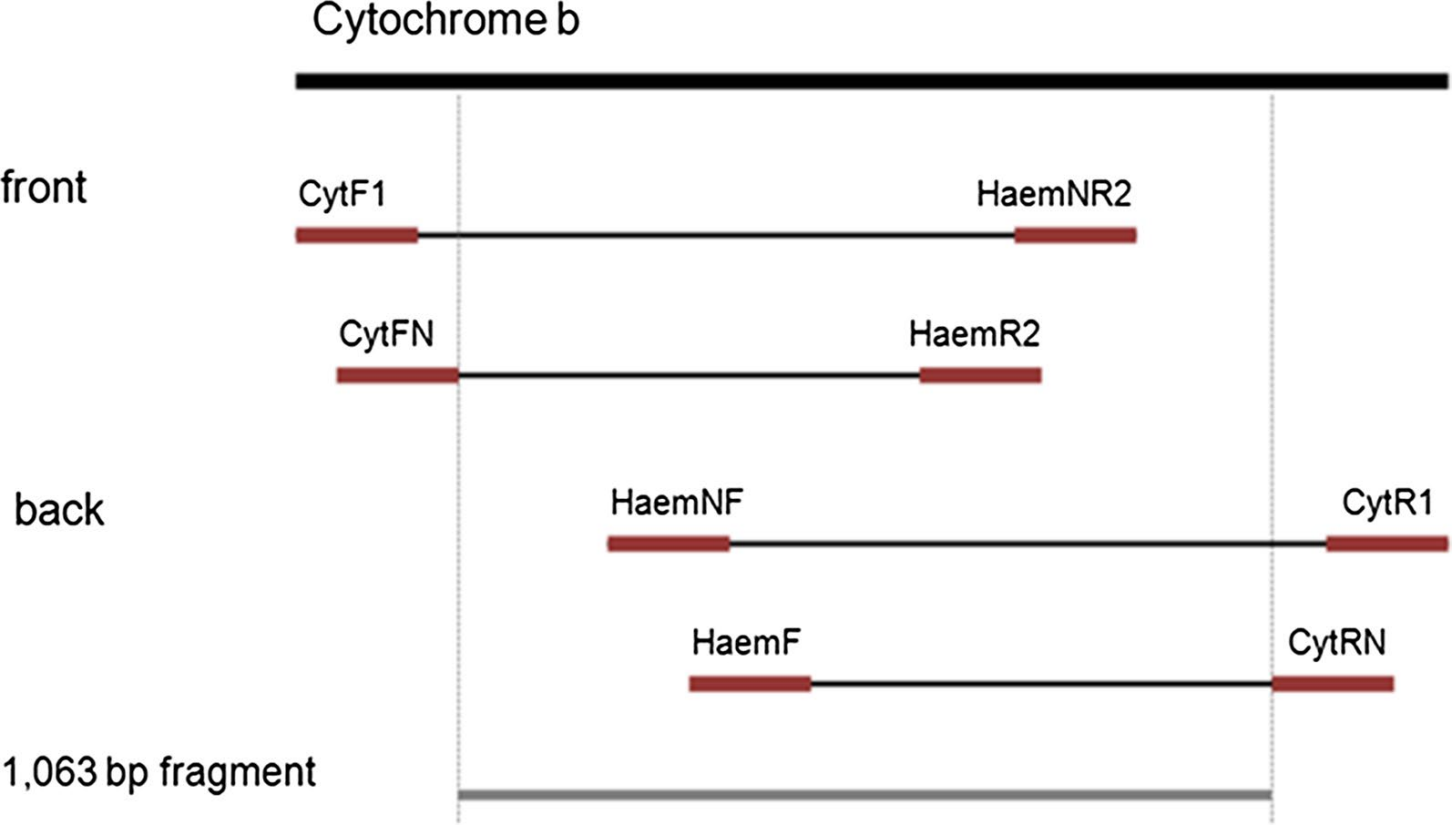
Schematic structure of PCRs used. PCRs amplify two separate pieces (front and back) of the haemosporidian cytochrome b gene. Red bars: primer location

After amplification, 5 μl of the PCR products stained with GelRed™ (BIOTREND, Köln, Germany) were visualized in a 1.5% agarose gel. In case of negative results, the authors tried to improve the sensitivity of the method by amplifying the 1063 bp fragment of the cytochrome *b* gene in two separate, overlapping fragments (Fig. 1). The reaction mixture for all PCRs was similar to the mixture described above. The cycling conditions for the primer sets CytF1/HaemNR2 and CytFN/HaemR2 were: 40 cycles 94 °C denaturation for 30 s, 52 °C annealing for 30 s and 72 °C elongation for 60 s. Cycling conditions for the primers HaemNF/CytR1 and HaemF/CytRN were identical except for the annealing temperature 50 °C for HaemNF/CytR1 and 54 °C for HaemF/CytRN.

All PCRs were primarily designed to amplify the cytochrome *b* gene of *Plasmodium* and *Haemoproteus* spp. The PCRs used for the “front” part of the fragment amplified *Leucocytozoon* spp., too. Therefore, we established the nested PCR primer LeucoF (5′-CCCATTCGTAGCATTAGC-3′) to amplify the “back” part of the target fragment of *Leucocytozoon* spp. Based on the amplification product of the PCR with the primers HaemNF and CytR1, we run a *Leucocytozoon* nested PCR with the primers LeucoF and CytRN. While the reaction mixture of this PCR was the same as described above, the cycling conditions were as follows: 40 cycles 94 °C denaturation for 30 s, 52 °C annealing for 30 s and 72 °C elongation for 60 s.

Each amplification product obtained from the second PCR step was visualized as described above. The amplification products were then purified using the PCR Product Purification Kit (Roche, Mannheim, Germany) and after sequencing (GATC Biotech AG) the resulting sequences were verified and separate fragments (“front” and “back”) were combined. All sequences were edited in GENtle (by Magnus Manske, University of Cologne, Germany). After trimming primers, a fragment of 1063 bp was obtained. The sequence dataset was aligned by eye in Multalin [25] and condensed to eliminate redundant sequences. Resulting sequences were then identified by matching sequences in MalAvi [11] and GenBank [26] using nucleotide BLAST search. Sequences showing double peaks were re-examined. By amplifying the “front” and “back” of the 1063 bp fragment it was possible to detect multiple haemosporidian infections which would not have been possible with previously published PCR methods. In case of no clear chromatogram, only the parasite genus was distinguished for the evaluation of the prevalence.

Any unique sequence, differing by one or more nucleotide from other recorded sequences, was considered to be a distinct lineage. Due to the length of the fragment used in this study, none of the detected sequences were similar to previously published sequences. All new sequences were deposited in GenBank (accession numbers MF189958–MF189970). The code of cytochrome b lineages is composed as follows: Pp (*Pica pica*) and/or Cc (*Corvus corone*) depending on the host followed by a letter “P” (*Plasmodium*), “H” (*Haemoproteus*) or “L” (*Leucocytozoon*) depending on the parasite genus.

### Statistical and phylogenetic analysis

The association among sex or age and the presence of parasite DNA was evaluated using the Chi squared test for independence (χ^2^ test, [27]). The dataset used for phylogenetic reconstruction consisted of 13 haemosporidian lineages obtained in this study and five reference sequences of different Haemosporida species downloaded from GenBank, each trimmed to 1063 bp to ensure consistency of sequence length. A cytochrome *b* sequence from *Theileria annulata* (KF732030.1) was used as outgroup. The dataset was analysed in MrModeltest v2.3 [28] to determine which nucleotide substitution model was appropriate based on the Akaike Information Criterion [29]. Phylogenetic analyses were performed using Bayesian inference performed in MrBayes v3.2.6 [30]. The HKY + G model was implemented and two Markov chains were run simultaneously for 50 million generations; trees were sampled every 1000 generations, resulting in 50,000 trees. 25% of the trees were discarded as “burn-in” period. The remaining trees were used to construct a majority rule consensus tree and to calculate posterior probabilities. To estimate bootstrap values and to check for congruence of phylogenetic relationships across multiple approaches, a maximum likelihood (ML) approach was implemented in MEGA v6 [31]. A phylogeny was generated implementing the general-time-reversible model (GTR + G) using 1000 replicates. The Bayesian majority consensus tree and maximum likelihood phylogram were viewed and edited with FigTree v1.4.3 (Andrew Rambaut, University of Edinburgh, England) and MEGA v6. The sequence divergence between different lineages and specified groups was calculated using Jukes-Cantor model of substitution implemented in the program MEGA v6.

TCS v1.21 [32] was used for a statistical parsimony network approach. Only the haplotypes of the “*Leucocytozoon fringillinarum*—clade” were used for this network to untangle this cryptic clade. To compare the cryptic clade found in this study with the one described by Freund et al. [20] another phylogeny was generated using MEGA v6, implementing the GTR + G model and using 1000 replicates. The dataset used for this approach consisted of the lineages forming the “*Leucocytozoon fringillinarum*—clade”, 10 *Leucocytozoon* lineages isolated from three corvid species from North America (American Crow *Corvus brachyrhynchos*, Yello-billed Magpie *Pica nutalli* and Stellers Jay *Cyanocitta stelleri*) by Freund et al. [20], *Plasmodium relictum* strain SGS1 (LN835311.1), *Haemoproteus minutus* (DQ630013.1) and *Theileria annulata* (KF732030.1) as outgroups.

## Results

Ninety-five liver samples of carrion crows were tested for the presence of haemosporidian DNA. Parasite DNA was successfully isolated from 85 samples (89.5%). Eighty-one samples (85.3%) were found positive for *Leucocytozoon* spp., 28 (29.5%) for *Plasmodium* spp. and 7 (7.4%) for *Haemoproteus* spp. This difference in prevalence of the different Haemosporida genera was pronounced (χ^2^ = 126.857; P < 0.0001), with the highest relative risk for *Leucocytozoon* spp.

**Fig. 2:**
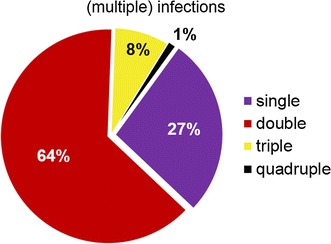
Amount of (multiple) haemosporidian infections found in carrion crows of southwest Germany. Most common was the occurrence of double infections (n = 54), followed by single infections (n = 23) and triple infections (n = 7). One quadruple infection was detected

Sixty-two samples (65.3%) contained multiple parasite lineages (Fig. 2). Most abundant was the double infection with *Leucocytozoon* and *Plasmodium* (n = 25) followed by a double infection of two different *Leucocytozoon* lineages (n = 18). Relatively rare were double infections with *Leucocytozoon* and *Haemoproteus* (n = 6) and only one double infection with *Plasmodium* and *Haemoproteus* was detected.

Carrion crows infected with haemosporidian parasites were uniformly distributed in the three observed age classes (juvenile n = 31, second year n = 33 and older than second year n = 31; χ^2^ = 1.83, P = 0.401), whereas female carrion crows were more often infected with haemosporidian parasites than male crows (males n = 42, females n = 49; χ^2^ = 5.18, P = 0.023) (Table 2).

**Table 2:** Sex and age distribution

	*Leucocytozoon* spp. prevalence % (n)	*Plasmodium* spp. prevalence n (%)	*Haemoproteus* spp. prevalence n (%)
Sex			
Male (n = 42)	78.6 (33)	31 (13)	7.1 (3)
Female (n = 49)	89.8 (44)	28.6 (14)	8.2 (4)
Age class			
<2 year (n = 31)	87.1 (27)	25.8 (8)	6.5 (2)
2nd-yearold (n = 33)	78.8 (26)	27.3 (9)	9.1 (3)
>2 year (n = 31)	90.3 (28)	35.5 (11)	6.5 (2)

Distribution of carrion crows tested positive by PCR for haemosporidian parasites in southwest Germany (collected in 2016)

Each of the five Eurasian Magpie liver tissue samples was tested positive for the presence of haemosporidian DNA. Two lineages of *Leucocytozoon* spp., Pp_L1 (n = 1) and Pp_L2 (n = 2), could be identified. While we found no sequence identical to lineage Pp_L1 (MF189969), Pp_L2 (MF189970) was found to be homologous to PICPIC01 (MalAvi database), which refers to an unpublished sequence isolated from a magpie from Turkey. Two Eurasian Magpie samples contained DNA of a lineage 99% identical with the *Plasmodium relictum* strain SGS1 (GenBank number LN835311.1). In addition, seven *Leucocytozoon* lineages (Cc_L1–L7), three *Plasmodium* (Cc_P1, Cc_P2 and Cc_Pp_P) and one *Haemoproteus* lineage (Cc_H) were identified among the carrion crows (Table 3).

**Table 3:** Parasite lineages found in this study

GenBank no.	Lineage	n (female, male)	% match	GenBank (1063 bp)	% match	MalAvi
MF189962	Cc_L1	10 (8, 2)	96	*L. fringillinarum* GenBank: FJ168564.1 *Pipilo chlorurus*	*100*	*Leucocytozoo*n sp. 4 FES-2014 (479 bp) GenBank: KJ128991.1 *C. cornix cornix* (Italy)
MF189963	Cc_L2	59 (21, 34)	95		*100*	*Leucocytozoon* sp. *C. corone* 6 (522 bp) GenBank: AB741497.1 *C. corone* (Japan)
MF189964	Cc_L3	24 (10, 13)	96		*100*	*Leucocytozoon* sp. JvR-2012 isolate COCOR3 (476 bp) GenBank: JX867112.1 *C. corone* (Switzerland) *Leucocytozoon* sp. *C. macrorhynchos* 1 (522 bp) GenBank: AB741500.1 *C. macrorhynchos* (Japan)
MF189965	Cc_L4	6 (2, 4)	95		99	*Leucocytozoon* sp. *C. corone* 6 (522 bp)
MF189966	Cc_L5	4 (2, 2)	96		99	*Leucocytozoon* sp. 2 FES-2014 (479 bp) GenBank: KJ128989.1 *C. cornix cornix* (Italy)
MF189967	Cc_L6	1 (0, 1)	97		99	*Leucocytozoon* sp. *C. macrorhynchos* 1 (522 bp)
MF189968	Cc_L7	1 (0, 1)	96		99	*Leucocytozoon*sp. *C. macrorhynchos 1* (522 bp)
MF189958	Cc_Pp_P	26 (12, 13)	99	*P. relictum* strain SGS1 GenBank: LN835311.1	*100*	*Plasmodium* sp. isolate SGS1 GenBank: AF495571.1 *Passer luteus* (Nigeria)
MF189959	Cc_P1	1 (0, 1)	99		*100*	*Plasmodium* sp. GRW11 (479 bp) GenBank: AY831748.1 *Sylvia atricapilla*
MF189960	Cc_P2	1 (1, 0)	98	*P. lutzi* isolate UN117A GenBank: KC138226.1 *Turdus fuscater* (Colombia)	*100*	*Plasmodium* sp. AFTRU5 (479 bp) GenBank: DQ847263.1
MF189961	Cc_H	7 (3, 4)	97	*H. minutus* isolate L-TURDUS2 GenBank: DQ630013.1 *Turdus merula* (Lithuania)	*100*	*Haemoproteus* sp. hCIRCUM05 (478 bp) GenBank: KC994900.1 *Culicoides circumscriptus* (Spain)

Matching results of haemosporidian lineages isolated from carrion crows (*Corvus corone*) of southwest Germany comparing data from GenBank (1063 bp) with data from the MalAvi database (sequences <522 bp). The number of males and females is given as (m, f). Support for lineages with no percentage was 100%

**Fig. 3:**
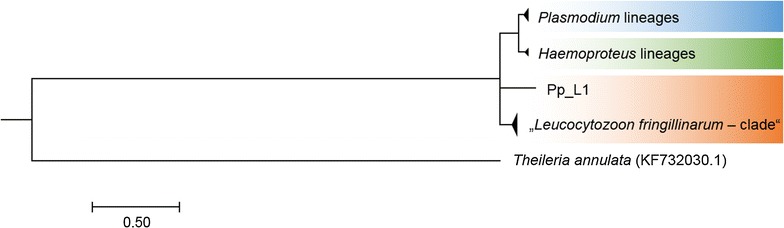
Phylogenetic tree of lineages obtained in this study combined with lineages from GenBank. Bayesian major consensus tree (constructed using MrBayes v3.2.6) based on 1063 bp of the cytochrome b gene using the HKY + G model. The lineages are shown in condensed clades representing the three genera of Haemosporida

**Fig. 4:**
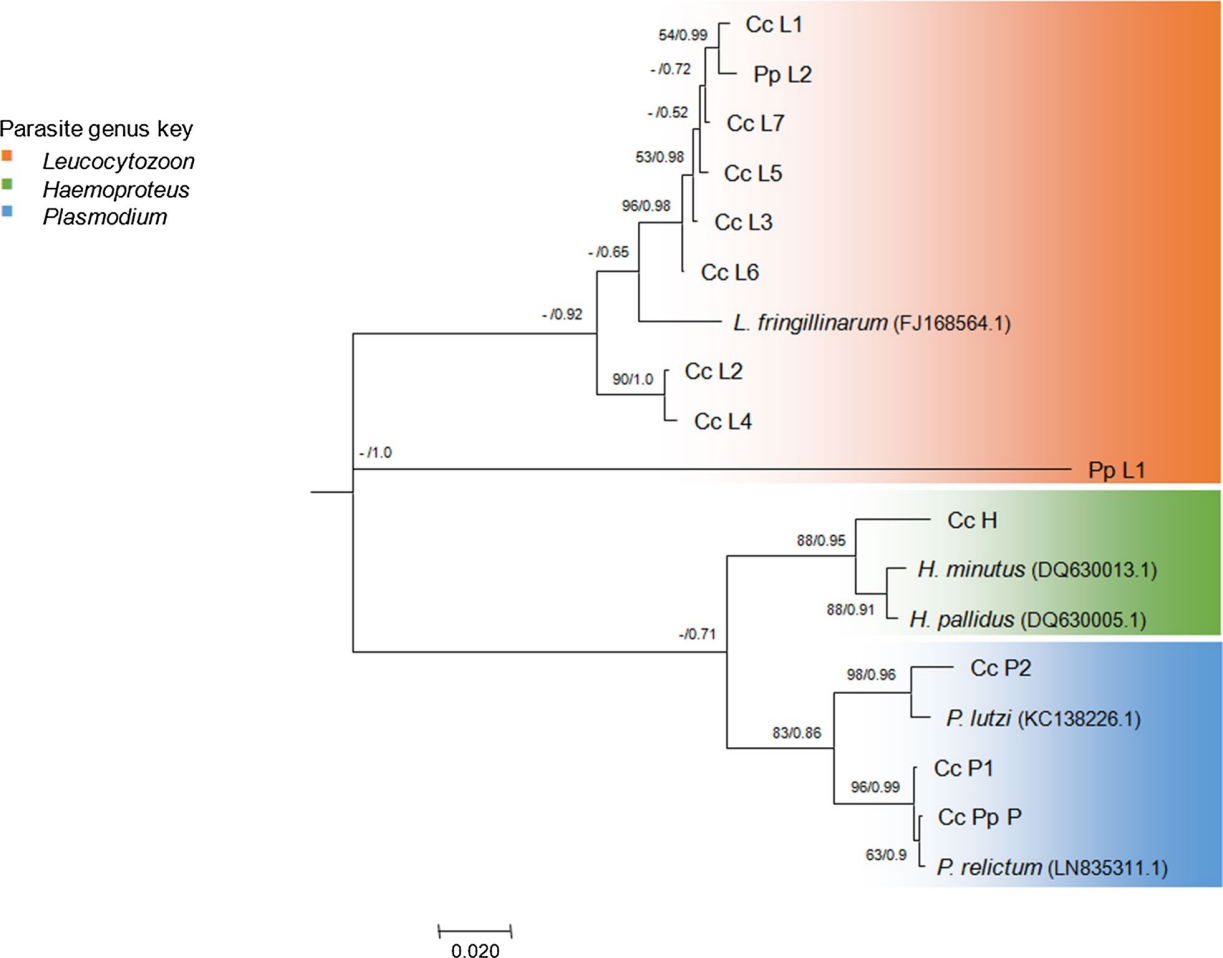
Subtree of Bayesian major consensus tree. Data are from two phylogenetic analyses. The visualized phylogeny is constructing using a Bayesian interference performed in MrBayes v3.2.6. The posterior probability is shown as the denominator on the branches. When the Bayesian major consensus tree was congruent with the constructed maximum-likelihood approach implemented in MEGA v6, the bootstrap support is shown as the nominator. GenBank accession numbers are noted behind the lineage names. The colours mark the different parasite genera the lineages belong to (orange: *Leucocytozoon*, green: *Haemoproteus*, blue: *Plasmodium*)

The construction of phylogenetic trees using MrBayes or MEGA v6 revealed similar results. A rooted phylogenetic tree of the lineages obtained in this study was combined with previously published sequences (GenBank; Fig. 3). The subtree with the haemosporidian parasite lineages is shown in Fig. 4. The Haemosporida genera are clearly separated. The *Leucocytozoon* lineage Pp_L1 isolated from one Eurasian Magpie is extremely distant from all the other lineages and matched only 80% with *Leucocytozoon fringillinarum* (FJ168564.1) using NCBI nucleotide BLAST search.

**Fig. 5:**
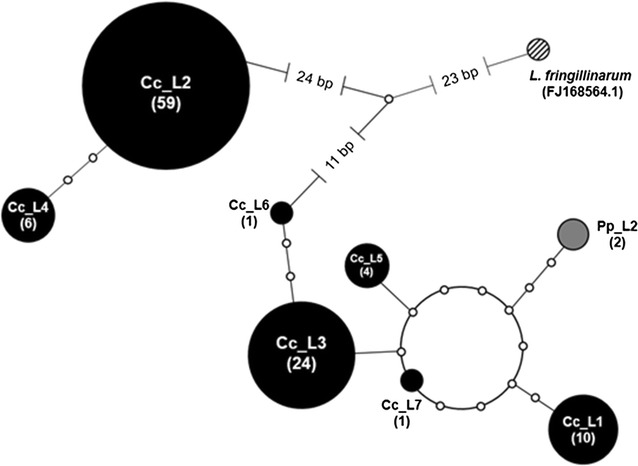
Haplotype network of “*Leucocytozoon fringillinarum—*clade” found in corvids from southwest Germany based on 1063 bp fragment of cytochrome b gene. The colours mark the different host species the lineages were isolated from (black: *Corvus corone*, grey: *Pica pica,* striped: *Pipilo chlorurus*). Number of isolates in brackets

Lineages were grouped (Table 4) according to the eight clusters present in the tree. Sequence divergence within and between the groups was calculated. The genetic divergence between the groups of the “*Leucocytozoon fringillinarum*—clade” (group 1–3) was ≤5%. The relation between the groups 1, 2 and 3 (shown in Table 4) was explored in a haplotype network (Fig. 5). The mean distance between the groups was 40 bp changes. Lineage Cc_L2 was the most abundant lineage found (n = 59) and Cc_L3 was the most abundant lineage in group 1 (n = 24).

**Table 4:** Sequence divergence of lineages obtained from corvids of southwest Germany and lineages obtained from GenBank

Cytochrome *b* sequence (0–1063 bp)										
Group		Mean between-group distance							No. of lineages	Mean within-group
		(1)	(2)	(3)	(4)	(5)	(6)	(7)		
(1)	Pp_L2, Cc_L1, Cc_L3, Cc_L5 – L7								6	0.0021
(2)	Cc_L2, Cc_L4	0.04							2	0.001
(3)	*L. fringillinarum* (FJ168564.1)	0.038	0.05						1	–
(4)	*H. minutus* (DQ630013.1), *H. pallidus* (DQ630005.1)	0.223	0.216	0.213					2	0.00244
(5)	Cc_H	0.238	0.225	0.228	0.029				1	–
(6)	*P. lutzi* (KC138226.1), Cc_P2	0.22	0.217	0.22	0.094	0.099			2	0.0056
(7)	*P. relictum* (LN835311.1), Cc_P1, Cc_Pp_P	0.225	0.221	0.22	0.088	0.048	0.048		3	0.00046
(8)	Pp_L1	0.315	0.313	0.3	0.346	0.365	0.369	0.353	1	–

Mean sequence divergence between and within different groups of Haemosporida determined after phylogenetic tree calculated for a 1063 bp fragment of cytochrome busing MEGA v6

## Discussion

Prevalence of haemosporidian parasites was high in carrion crows (89.5%, n = 95) and all five Eurasian Magpies were infected. *Leucocytozoon* spp. proved to be the most prevalent parasite genus in carrion crows (n = 81), followed by *Plasmodium* spp. (n = 28) and *Haemoproteus* spp. (n = 7). These findings are in agreement with Scaglione et al. [19] who found 97.9% (46 out of 47) prevalence of *Leucocytozoon* spp. in hooded crows (*Corvus corone cornix*). The high prevalence of *Leucocytozoon* spp. compared to the related parasite genus *Plasmodium* may reflect a more restricted host range for the hematophagous vectors. Whereas vector species of other Haemosporida may be more strictly ornithophilic, the vectors of avian *Plasmodium* spp. may feed on a wider variety of vertebrate fauna, reducing their vector potential [33]. The low prevalence of *Haemoproteus* spp. might be due to a lack of suitable vectors in the study area or a successful adaptation of the birds to the parasite. Scaglione et al. [19] discussed that the high prevalence of avian haematozoa in hooded crows emphasizes the success of ornithophilic vectors and the susceptibility of corvids to infection.

Co–infections drive evolution of virulence. The selection pressure imposed by parasites on the host is expected to be stronger when several parasites exploit the same host [34]. Studies based on microscopic examination of blood smears [35, 36] as well as a few studies using molecular diagnostic methods [14] have shown that mixed infections are common in many bird–parasite systems. The use of conventional PCR assays underestimates biodiversity of haemosporidian parasites because they fail in detecting multiple infections [37]. By combining different sets of PCR methods, we found 65.3% (n = 95) multiple infections which is remarkable. Inevitably this leads to the question how the birds can cope with this. Using data from a natural population of house martins (*Delichon urbicum*) Marzal et al. [38] tested whether the infection with two malaria parasite lineages had more negative effects than a single infection. Their findings demonstrated that double malaria infections decreased survival. The crows used in this study were lured by a dummy in their natural habitat. Therefore, it is assumed that all the crows shot were probably in good physical condition although they suffered from parasite infection. This might be due to an evolutionary adaptation between crows and their avian blood parasites.

The majority of studies about age effects on haemosporidian prevalence indicate that adult birds have a greater prevalence but in some studies prevalence is higher in young birds, while the remainder find no differences [1]. Prevalence of bird infections among the various age groups of *Corvus corone* was similar in this study, which is in accordance with the results of Hooded Crows *Corvus cornix* [19]. In a study of Burkett-Cadena et al. [39] about vector-host interactions in avian nests neither nestlings nor adult birds were preferred for a blood meal by mosquitoes.

Female carrion crows showed a higher prevalence in haemosporidian parasites than males. The decrease of locomotion activity of birds during the nesting period is a factor increasing the probability of their infection with haemosporidians [1]. Female carrion crows perform all incubation and brooding and pluck out their feathers on the breast creating a naked and swollen brood patch only poorly covered by feathers [40]. They may, therefore, be more exposed to vectors of haemosporidian parasites.

In this study, DNA of 13 different haemosporidian parasite lineages was isolated. Two lineages were exclusively from Eurasian Magpies, 10 from carrion crows and one *Plasmodium* lineage was isolated from both bird species. Due to differences in the length of gene fragments deposited in GenBank and the MalAvi database, the comparison of our sequences was difficult, especially in the case of the isolated *Leucocytozoon* lineages. All *Leucocytozoon* sequences in our study showed the highest consensus with *Leucocytozoon fringillinarum* (FJ168564.1) isolated from *Pipilo chlorurus* (Passerellidae), but the homology calculated by NCBI BLAST search was merely in the range of 80% for Pp_L1 to 97% for Cc_L6. Within the MalAvi database eight sequences were 100% similar to those found in this study (Table 3), however, these sequences have a length of only up to 522 bp. Since our 1063 bp fragment of cytochrome *b* has more than double the length of the sequences of the MalAvi database it is possible that the compared sequences are not 100% identical throughout the whole 1063 bp fragment. This issue should be further examined.

**Fig. 6:**
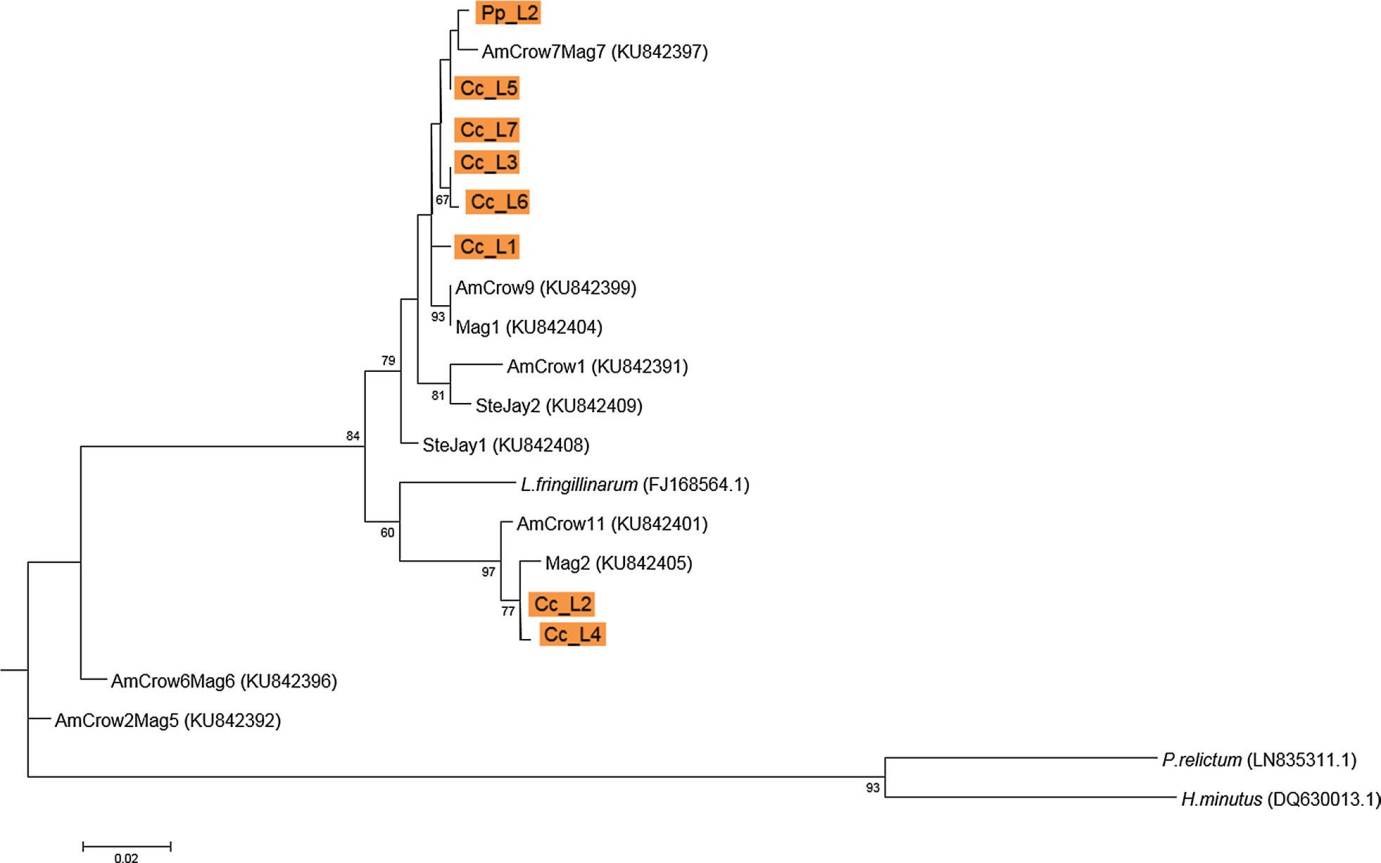
Phylogenetic tree of lineages from Germany combined with lineages from Freund et al. [20] and additional ones from GenBank. Maximum likelihood model based on 1063 bp of haemosporidian cytochrome b gene constructed using a GTR + G model of nucleotide substitution. Numbers at the nodes represent bootstrap support values above 50%, based on 1000 replicates. Lineages highlighted in orange were obtained in this study. Germany: Cc = *Corvus corone*, Pp = *Pica pica*, California: AmCrow = C*orvus brachyrhynchos*, Mag = *Pica nutalli* and SteJay = *Cyanocitta stelleri*

The *Leucocytozoon* lineages found in this study (Cc_L1–L7) were similar or nearly homologous (99%) to the short sequences (<522 bp Cyt b fragment) of *Leucocytozoon* spp. previously isolated from *Corvus* spp. either from Italy, Japan or Switzerland (MalAvi database). It was reported that *Leucocytozoon berestneffi* and *Leucocytozoon sakharoffi* are the most important haemosporidian parasites for corvids [20]. Current taxonomic classification indicates intra-family host specificity with *Leucocytozoon sakharoffi* infecting crows and related *Corvus* spp. as well as *Leucocytozoon berestneffi* infecting magpies (*Pica* spp.) and blue jays (*Cyanocitta* spp.) [1]. However, recent phylogenetic reconstructions revealed a single large clade containing nearly every lineage recovered from the three host species of Northern-America and Japan, while showing no evidence of the expected distinction between *Leucocytozoon sakharoffi* and *Leucocytozoon berestneffi.* It was suggested that the two parasite lineages are in fact part of one large corvid-parasite complex [20]. Combining the *Leucocytozoon* sequences obtained from our study with ten lineages found in North-American corvids [20] showed that the lineages from Germany are also part of the corvid-parasite complex mentioned above (Fig. 6). *Corvus* spp. presumably radiated in the western Palearctic (Europe) and later immigrated to the new world whereas magpies and new world jays speciated after immigration to the new world [41, 42]. Data on more host species are needed to interpret: 1. how parasite phylogeny could relate to bird host phylogeny, 2. whether lineages found so far show host specificity for corvids and 3. if the mentioned corvid-parasite complex is present worldwide.

The Biological Species concept is difficult to evaluate in malaria parasites and most researchers have used variations of morphological and phylogenetic species concepts [12, 43]. In this study, the genetic divergence was described to draw initial conclusions as to whether lineages we found may be species or haplotypes as proposed by Outlaw and Ricklefs [43]. Including samples of this study and homologous lineages previously published in GenBank, eight groups were classified according to the clusters present in the phylogenetic tree and the mean sequence divergence between and within the different groups was determined. This revealed the basal position of *Leucocytozoon* spp. as already known from other studies (e.g. [44–46]. However, the *Leucocytozoon* lineage Pp_L1 (group 8), isolated from a single *Pica pica*, has a very special position. It is clearly separated from all other groups and shows a high sequence divergence of >30%. According to Hellgren et al. [47] a mean genetic variation of 5.5% indicates that these lineages are likely to be morphologically distinct. Therefore, it is proposed that the lineage *Leucocytozoon* sp. Pp_L1 might be a new species. Due to the fact that this putative species differs so much from all other published parasite sequences, it is assumed that it has probably another natural host or vector that has not yet been investigated.

The so called “*Leucocytozoon fringillinarum*-clade” consists of three groups (1, 2, 3). The mean sequence divergence between the groups was 3.8–5%, the divergence within the groups 1 and 2 was <0.2%. Therefore, the two clusters seem to consist of numerous haplotypes and are part of the corvid-parasite complex mentioned above.

The *Haemoproteus* lineage Cc_H (group 5) isolated from seven carrion crows in our study shows genetically a clear separation from the *Leucocytozoon* and *Plasmodium* groups (1–3 and 6–8) with sequence divergences more than 22.5%. However the divergence between group 5 and 4 [*Haemoproteus minutus* (DQ630013.1), *Haemoproteus pallidus* (DQ630005.1)] is only 2.9%. Hellgren et al. [47] found the morphologically distinct species (*Haemoproteus pallidus* and *Haemoproteus minutus*) showing a genetic distance of only 0.7% (in our study 0.244%). Thus it is unclear whether the lineage *Haemoproteus* sp. Cc_H might represent its own species. We found a 97% consensus with the lineage *Haemoproteus minutus* (DQ630013) isolated from *Turdus merula* from Lithuania [47] deposited in GenBank. However, in MalAvi a sequence identical to our lineage Cc_H, *Haemoproteus* sp. hCIRCUM05 (KC994900) isolated from *Culicoides circumscriptus* (Ceratopogonidae) from Spain [48] was found. Although this published sequence has a length of only 478 bp, it could still represent the same parasite species. According to the IIKC (Interactive Identification Key for Culicoides (Diptera: Ceratopogonidae) from the Western Palaearctic region [49]) *Culicoides circumscriptus* is also present in Germany. Therefore, it is hypothesized that there might be a lifecycle of *Haemoproteus* sp. hCIRCUM05/Cc_H with carrion crows as host and *Culicoides circumscriptus* as a vector. However, this has to be confirmed by analyses of blood-fed biting midges from infection experiments.

The *Plasmodium* lineage Cc_P2 and *Plasmodium lutzi* (KC138226.1) forming group 6 differ in 17 of 1063 bp, representing a sequence divergence of 0.56%. This could be due to intraspecific variation and the lineages actually might be morphologically identical. Group 7 consists of *P. relictum* (LN835311.1), Cc_P1 and Cc_Pp_P. The lineage Cc_Pp_P which has been isolated from both carrion crows and Eurasian Magpies was similar to the previously reported *Plasmodium* lineage SGS1 (e.g. [50–52]). The lineage Cc_P1 was identical with the previously published lineage GRW11 (e.g. [53, 54]). *Plasmodium* sp. SGS1 and GRW11 have been previously isolated from different birds (Passeriformes and Non-Passeriformes) and from mosquitoes. Since these lineages have already been isolated several times from different bird species in different regions, one could assume that these parasites are generalists. Okanga et al. [50] stated that e.g. SGS1 may have come to Africa in migratory birds from Europe. The reported lineages differ only in one single nucleotide. Therefore, the lineages Cc_P1 and Cc_Pp_P seem to be two newly detected haplotypes of the numerous lineages forming the morphological parasite species of *Plasmodium relictum* (after [15]). Future studies should reveal whether the lineage *Plasmodium* sp. Cc_Pp_P found in this study is a strain restricted to Germany or is specialized on corvids.

## Conclusion

The prevalence of carrion crows and Eurasian Magpies infected with haemosporidian parasites was very high with *Leucocytozoon* spp. being the most abundant taxon. Due to the applied diagnostic method, it was possible to detect a high amount of multiple infections. Data of this study suggest that female carrion crows are more likely to be infected with haemosporidian parasites than males. 13 different Haemosporida lineages were detected and examined. In future studies morphological and phylogenetic species concepts should be combined to test proposed species delimitations as outlined by Outlaw and Ricklefs [43].

## References

[1] Valkiunas G (2005). Avian malaria parasites and other Haemosporidia.

[2] Santiago-Alarcon D, Palinauskas V, Schaefer HM (2012). Diptera vectors of avian Haemosporidian parasites: untangling parasite life cycles and their taxonomy. Biol Rev.

[3] James SP, Tate P (1938). Exo-erythrocytic schizogony in *Plasmodium gallinaceum* Brumpt, 1935. Parasitology.

[4] Cox FEG (2010). History of the discovery of the malaria parasites and their vectors. Parasit Vectors..

[5] Clark NJ, Clegg SM, Lima MR (2014). A review of global diversity in avian haemosporidians (*Plasmodium* and *Haemoproteus*: Haemosporida): new insights from molecular data. Int J Parasitol.

[6] Beadell JS, Covas R, Gebhard C, Ishtiaq F, Melo M, Schmidt BK (2009). Host associations and evolutionary relationships of avian blood parasites from West Africa. Int J Parasitol.

[7] Bensch S, Perez-Tris J, Waldstrom J, Hellgren O (2004). Linkage between nuclear and mitochondrial DNA sequences in avian malaria parasites: multiple cases of cryptic speciation?. Evolution.

[8] Clark G (1965). Schizogony and gametocyte development of *Leucocytozoon berestnefi* in the Yellow-Billed Magpie, Pica nuttali. J Protozool..

[9] Bishop MA, Bennett GF (1990). The haemoproteids of the families Corvidae (crows and jays) and Sturnidae (starlings and mynas) (Passeriformes). Can J Zool.

[10] Bennett GF, Peirce MS (1992). Leucocytozoids of 7 old world passeriform familes. J Nat Hist..

[11] Bensch S, Hellgren O, Pérez-Tris J (2009). MalAvi: a public database of malaria parasites and related haemosporidians in avian hosts based on mitochondrial cytochrome b lineages. Mol Ecol Resour..

[12] Martinsen ES, Paperna I, Schall JJ (2006). Morphological versus molecular identification of avian Haemosporidia: an exploration of three species concepts. Parasitology.

[13] Sato Y, Hagihara M, Yamaguchi T, Yukawa M, Murata K (2007). Phylogenetic comparison of *Leucocytozoon* spp. from wild birds of Japan. J Vet Med Sci.

[14] Mata VA, da Silva LP, Lopes RJ, Drovetski SV (2015). The Strait of Gibraltar poses an effective barrier to host-specialised but not to host-generalised lineages of avian Haemosporidia. Int J Parasitol.

[15] Beadell JS, Ishtiaq F, Covas R, Melo M, Warren BH, Atkinson CT (2006). Global phylogeographic limits of Hawaii’s avian malaria. Proc R Soc B Biol Sci..

[16] Martínez-de la Puente J, Muñoz J, Capelli G, Montarsi F, Soriguer F, Arnoldi D, Rizzoli A (2015). Avian malaria parasites in the last supper: identifying encounters between parasites and the invasive Asian mosquito tiger and native mosquito species in Italy. Malar J..

[17] Kim KS, Tsuda Y (2010). Seasonal changes in the feeding pattern of *Culex pipiens pallens* govern the transmission dynamics of multiple lineages of avian malaria parasites in Japanese wild bird community. Mol Ecol.

[18] Leclerc A, Chavatte JM, Landau I, Snounou G, Petit T (2014). Morphologic and molecular study of hemoparasites in wild corvids and evidence of sequence identity with *Plasmodium* DNA detected in captive black-footed penguins (*Spheniscus demersus*). J Zoo Wildl Med..

[19] Scaglione FE, Cannizzo FT, Pregel P, Perez-Rodriguez AD, Bollo E (2016). Blood parasites in hooded crows (*Corvus corone cornix*) in Northwest Italy. Vet Ital..

[20] Freund D, Wheeler S, Townsend AK, Boyce WM, Ernest HB, Cicero C (2016). Genetic sequence data reveals widespread sharing of *Leucocytozoon* lineages in corvids. Parasitol Res.

[21] Bairlein F, Dierschke J, Dierschke V, Salewski V, Geiter O, Hüppop K (2014). Atlas des Vogelzugs.

[22] Demongin L (2016). Identification guide to birds in the hand.

[23] Waldenström AJ, Bensch S, Hasselquist D, Östman Ö (2004). A new nested polymerase chain reaction method very efficient in detecting *Plasmodium* and *Haemoproteus* infections from avian blood. J Parasitol.

[24] Bensch S, Stjernman M, Hasselquist D, Ostman O, Hansson B, Westerdahl H (2000). Host specificity in avian blood parasites: a study of *Plasmodium* and *Haemoproteus* mitochondrial DNA amplified from birds. Proc R Soc B Biol Sci..

[25] Corpet F (1988). Multiple sequence alignment with hierarchial clustering. Nucleic Acids Res.

[26] Benson DA, Cavanaugh M, Clark K, Karsch-Mizrachi I, Lipman DJ, Ostell J (2013). GenBank. Nucleic Acids Res.

[27] Preacher KJ. Calculation for the Chi square test: An interactive calculation tool for Chi square tests of goodness of fit and independence. 2001. http://quantpsy.org. Accessed 14 Feb 2017.

[28] Nylander JAA (2004). MrModeltest v2. Program distributed by the author. Evol Biol Cent Uppsala Univ..

[29] Huelsenbeck JP, Crandall KA (1997). Phylogeny estimation and hypothesis testing using maximum likelihood. Annu Rev Ecol Syst.

[30] Ronquist F, Huelsenbeck JP (2003). MrBayes 3: Bayesian phylogenetic inference under mixed models. Bioinformatics.

[31] Tamura K, Stecher G, Peterson D, Filipski A, Kumar S (2013). MEGA6: molecular evolutionary genetics analysis version 6.0. Mol Biol Evol.

[32] Clement M, Posada D, Crandall KA (2000). TCS: a computer pragram to estimate gene genealogies. Mol Ecol.

[33] Savage AF, Robert V, Goodman SM, Raharimanga V, Raherilalao MJ, Andrianarimisa A (2009). Blood parasites in birds from Madagascar. J Wildl Dis.

[34] Arriero E, Møller AP (2008). Host ecology and life-history traits associated with blood parasite species richness in birds. J Evol Biol.

[35] Valkiūnas G, Iezhova TA, Shapoval AP (2003). High prevalence of blood parasites in hawfinch *Coccothraustes coccothraustes*. J Nat Hist..

[36] Palinauskas V, Markovets MY, Kosarev VV, Efremov VD, Sokolov LV (2005). Occurrence of avian haematozoa in Ekaterinburg and Irkutsk districts of Russia. Ekologija..

[37] Bernotiene R, Palinauskas V, Iezhova T, Murauskaite D, Valkiunas G (2016). Avian haemosporidian parasites (Haemosporida): a comparative analysis of different polymerase chain reaction assays in detection of mixed infections. Exp Parasitol.

[38] Marzal A, Bensch S, Reviriego M, Balbontin J, de Lope F (2008). Effects of malaria double infection in birds: one plus one is not two. J Evol Biol.

[39] Burkett-Cadena ND, Ligon RA, Liu M, Hassan HK, Hill GE, Eubanks MD (2010). Vector-host interactions in avian nests: do mosquitoes prefer nestlings over adults?. Am J Trop Med Hyg.

[40] Bolopo D, Canestrari D, Marcos JM, Baglione V (2015). Nest sanitation in cooperatively breeding Carrion Crows. Auk..

[41] Lee SI, Parr CS, Hwang Y, Mindell DP, Choe JC (2003). Phylogeny of magpies (genus *Pica*) inferred from mtDNA data. Mol Phylogenet Evol.

[42] Ericson PGP, Jansén AL, Johansson US, Ekman J (2005). Inter-generic relationships of the crows, jays, magpies and allied groups (Aves: Corvidae) based on nucleotide sequence data. J Avian Biol.

[43] Outlaw DC, Ricklefs RE (2014). Species limits in avian malaria parasites (Haemosporida): how to move forward in the molecular era. Parasitology.

[44] Perkins SL, Schall JJ (2002). A molecular phylogeny of malarial parasites recovered from cytochrome b gene sequences. J Parasitol.

[45] Martinsen ES, Perkins SL, Schall JJ (2008). A three-genome phylogeny of malaria parasites (*Plasmodium* and closely related genera): evolution of life-history traits and host switches. Mol Phylogenet Evol.

[46] Borner J, Pick C, Thiede J, Kolawole OM, Kingsley MT, Schulze J (2016). Phylogeny of haemosporidian blood parasites revealed by a multi-gene approach. Mol Phylogenet Evol.

[47] Hellgren O, Krizanauskiene A, Valkiunas G, Bensch S (2007). Diversity and phylogeny of mitochondrial cytochrome b lineages from six morphospecies of avian Haemoproteus (Haemosporida: Haemoproteidae). J Parasitol.

[48] Ferraguti M, Martínez-de la Puente J, Ruiz S, Soriguer R, Figuerola J (2013). On the study of the transmission networks of blood parasites from SW Spain: diversity of avian haemosporidians in the biting midge *Culicoides circumscriptus* and wild birds. Parasit Vectors..

[49] Mathieu B, Cêtre-Sossah C, Garros C, Chavernac D, Balenghien T, Carpenter S (2012). Development and validation of IIKC: an interactive identification key for *Culicoides* (Diptera: Ceratopogonidae) females from the Western Palaearctic region. ParasitVectors..

[50] Okanga S, Cumming GS, Hockey PR, Nupen L, Peters JL (2014). Host specificity and co-speciation in avian haemosporidia in the Western Cape, South Africa. PLoS One..

[51] Quillfeldt P, Martínez J, Hennicke J, Ludynia K, Gladbach A, Massello JF (2010). Hemosporidian blood parasites in seabirds—a comparative genetic study of species from Antarctic to tropical habitats. Naturwissenschaften.

[52] Inci A, Yildirim A, Njabo KY, Duzlu O, Biskin Z, Ciloglu A (2012). Detection and molecular characterization of avian *Plasmodium* from mosquitoes in central Turkey. Vet Parasitol.

[53] Pérez-Tris J, Bensch S (2005). Dispersal increases local transmission of avian malarial parasites. Ecol Lett.

[54] Kazlauskienė R, Bernotienė R, Palinauskas V, Iezhova TA, Valkiūnas G (2013). *Plasmodium relictum* (lineages pSGS1 and pGRW11): complete synchronous sporogony in mosquitoes *Culex pipiens pipiens*. Exp Parasitol.

